# One new species and one newly recorded species of the genus *Lasiochira* Meyrick, 1931 (Lepidoptera, Oecophoridae) from China

**DOI:** 10.3897/zookeys.918.48544

**Published:** 2020-03-12

**Authors:** Aihui Yin, Yanpeng Cai

**Affiliations:** 1 Morphological Laboratory, Guizhou University of Traditional Chinese Medicine, Guiyang, 550025, Guizhou, China Guizhou University of Traditional Chinese Medicine Guiyang China

**Keywords:** Checklist, key, morphology, moth, taxonomy

## Abstract

*Lasiochira wuzhishanensis* Yin, **sp. nov.** is described herein from the island province of Hainan, China. It is diagnosed from a similar species, *Lasiochira jianfengensis* Yin, Wang & Park, 2014. Both species are sympatric in Hainan province, but the latter is also known in northern Vietnam. *Lasiochira pallidiptera* Yin, Wang & Park, 2014 is recorded for the first time from China. Color images of the adults and genitalia of the above three species are provided. In addition, a checklist and a geographical distribution map of all species of *Lasiochira* Meyrick are included.

## Introduction

*Lasiochira* Meyrick, 1931 is a small genus included in the Oecophoridae, with unknown biology. The type species, *Lasiochira camaropa* Meyrick, 1931, was diagnosed based on two male syntype specimens from Kwanhsien, China. Clarke (1963) designated a lectotype from Meyrick’s original material and transferred a second species, *L. xanthacma* (Meyrick, 1938), from *Allotalanta* Meyrick, 1913. No further taxonomic work was published on the genus until Wang (2006) treated the Oecophoridae of China, including the two known species. Yin et al. (2014) described six new species, but the entire fauna is known only from China, Korea, and Vietnam.

**Figures 1–3.**
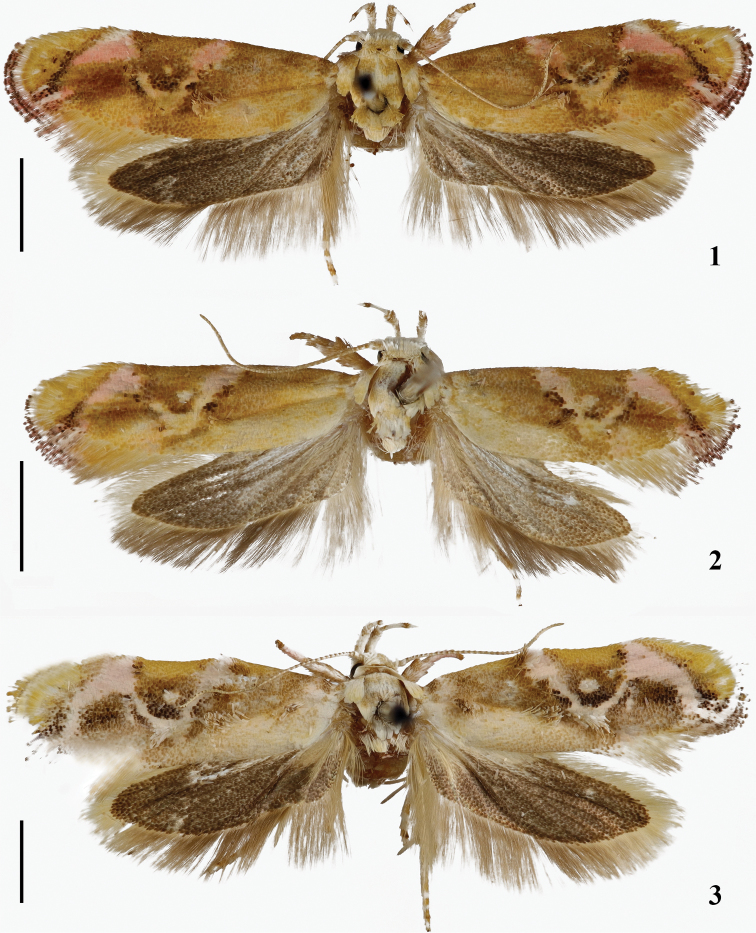
Adults of *Lasiochira* species **1***L. wuzhishanensis* Yin, sp. nov., paratype, female, gen. slide no. YAH19072 **2***L. jianfengensis* Yin, Wang and Park, female, gen. slide no. YC00028 **3***L. pallidiptera* Yin, Wang and Park, male, gen. slide no. YAH18154. Scale bars: 2.00 mm.

**Figures 4–7.**
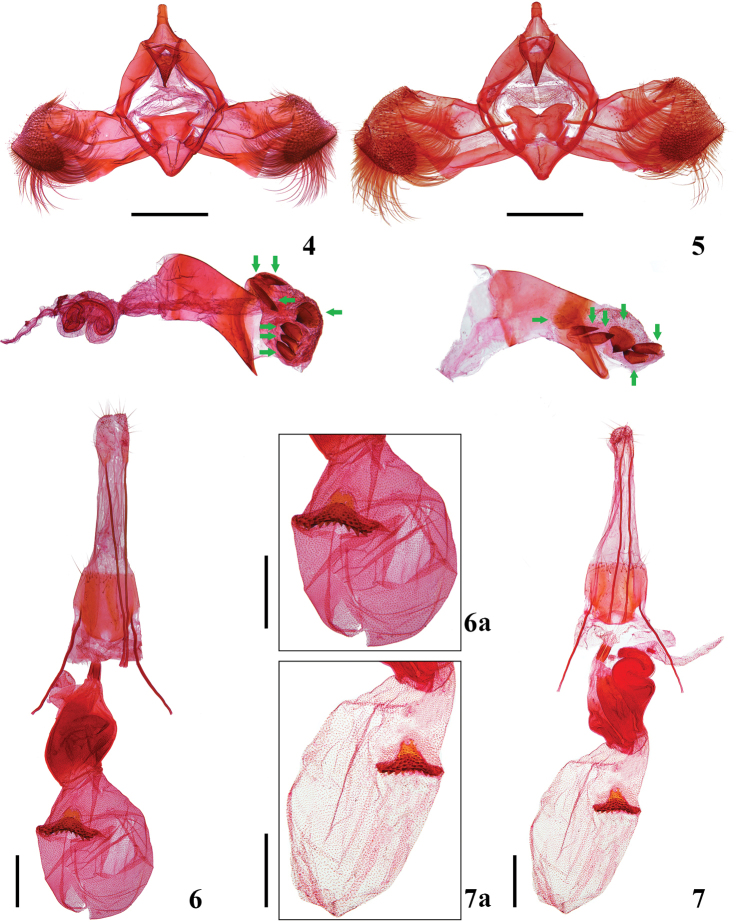
Male and female genitalia of *Lasiochira* species **4***L. wuzhishanensis* Yin, sp. nov., holotype, male genital capsule and phallus, gen. slide no. YC00025 **5***L. jianfengensis* Yin, Wang and Park, male genital capsule and phallus, gen. slide no. YC00024 **6***L. wuzhishanensis* Yin, sp. nov., paratype, female genitalia, gen. slide no. YC00023 **6a***L. wuzhishanensis* Yin, sp. nov., enlargement of corpus bursae, gen. slide no. YC00023 **7***L. jianfengensis* Yin, Wang and Park, female genitalia, gen. slide no. YC00028 **7a***L. jianfengensis* Yin, Wang and Park, enlargement of corpus bursae, gen. slide no. YC00028. Scale bars: 0.40 mm.

**Figures 8, 9.**
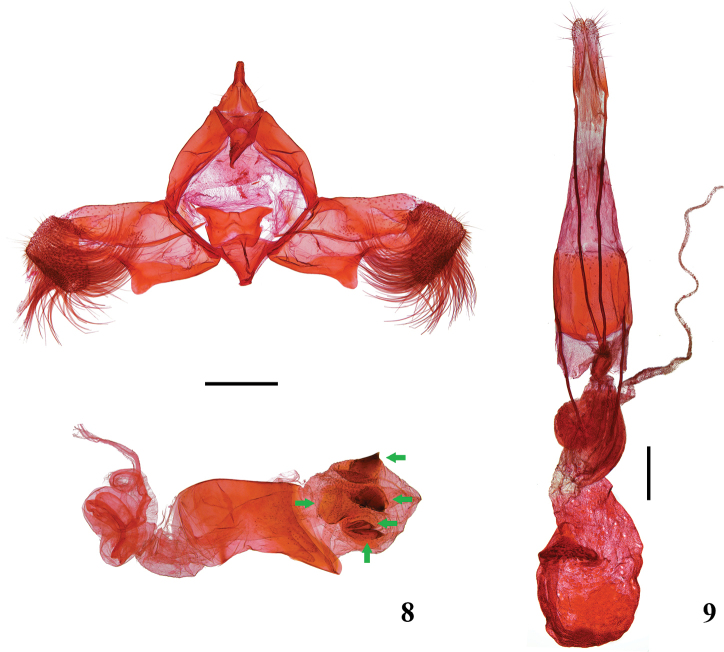
Male and female genitalia of *L. pallidiptera* Yin, Wang and Park **8** male genital capsule and phallus, gen. slide no. YAH19075 **9** female genitalia, gen. slide no. YAH18160. Scale bars: 0.40 mm.

The genus *Lasiochira* is mainly characterized by having a pale, N-shaped pattern consisting of three joined stripes and several small tufts of erect scales on the forewing (Figs 1–3); the gnathos is often pointed and hooked terminally (Figs 4, 5, 8); the valva usually has dense fine hairs on the distal half (Figs 4, 5, 8); the cornuti are comprised of multiple short, stout spines or plates (Figs 4, 5, 8; arrows); the apophyses posteriores are longer than the apophyses anteriores (Figs 6, 7, 9); the ductus bursae is with sclerotization (Figs 6, 7, 9); and the signum is transverse, dentate, with a projecting semicircular lobe posteriorly (Figs 6a, 7a, 9) (Yin et al. 2014).

Recent collecting efforts in the Hainan and Hubei provinces produced a new species, described herein, and the first record of *L. pallidiptera* Yin, Wang & Park, 2014 in China. The objectives of this study are to describe the new species and update the distributions of the known species.

## Material and methods

All specimens for this study were collected in 2018 from the Hainan and Hubei provinces of China. The descriptive terminology of the anatomical structures follows Wang (2006), Yin et al. (2014) and Kristensen (2003). Photographs of adults were taken using a Canon EOS 6D Mark II camera with an EF 100 mm f/2.8L MACRO IS USM lens assisted by the EOS Utility 3.10.20 software. Stacked images of the genitalia were captured using a Leica DM4 B upright microscope. Photomontage was performed with the Leica Application Suite X imaging software. Species distribution data were compiled within Microsoft Excel using both published records and specimen label data. The distribution map was produced with the aid of DIVA-GIS 7.5 (Hijmans et al. 2011).

All type specimens are deposited in the Morphological Laboratory, Guizhou University of Traditional Chinese Medicine, Guiyang, Guizhou, China.

## Taxonomy

### Lasiochira

Taxon classificationAnimaliaLepidopteraOecophoridae

Meyrick, 1931: 71.

4CD54479-4A7E-56E1-9298-E60E51832917

#### Type species.

*Lasiochira camaropa* Meyrick, 1931, by monotypy.

### Checklist of *Lasiochira* Meyrick species

1 *Lasiochira camaropa* Meyrick, 1931: 71

Distribution: China (Sichuan province).

2 *Lasiochira flaviterminata* Yin, Wang & Park, 2014: 33

Distribution: China (Chongqing City).

3 *Lasiochira jianfengensis* Yin, Wang & Park, 2014: 25

Distribution: China (Hainan province), Vietnam (North).

4 *Lasiochira jiulongshana* Yin, Wang & Park, 2014: 27

Distribution: China (Zhejiang province).

5 *Lasiochira pallidiptera* Yin, Wang & Park, 2014: 30

Distribution: China (Hubei province), Korea (Central).

6 *Lasiochira rosataenia* Yin, Wang & Park, 2014: 32

Distribution: Vietnam (North).

7 *Lasiochira taiwanensis* Yin, Wang & Park, 2014: 31

Distribution: China (Taiwan province).

8 *Lasiochira wuzhishanensis* Yin, sp. nov.

Distribution: China (Hainan province).

9 *Lasiochira xanthacma* (Meyrick, 1938: 10)

Distribution: China (Guangdong, Guizhou, Henan, Shaanxi, Shanxi and Yunnan provinces).

### Lasiochira wuzhishanensis

Taxon classificationAnimaliaLepidopteraOecophoridae

Yin sp. nov.

8A4727BB-B6B2-588F-B0AF-F4501BE5A0CE

http://zoobank.org/4001CC9D-E7DA-4374-9BF1-28A607ADAD91

Figs 1 , 4 , 6 , 6a

#### Material examined.

***Holotype***: ♂; China: Hainan province, Wuzhishan City, Wuzhishan National Nature Reserve; alt. 650 m; 18°54'36"N, 109°40'48"E; 10 May 2018; Zhengyong Wang leg.; YC00025. ***Paratypes***: 1 ♂, 3 ♀♀; same locality as holotype; alt. 650 m; 18°54'36"N, 109°40'48"E; 10–15 May 2018; Zhengyong Wang leg.; YC00026 ♂, YC00022 ♀, YC00023 ♀, YAH19072 ♀.

#### Diagnosis.

*Lasiochira wuzhishanensis* Yin, sp. nov. can be distinguished from its congeners by the following two character states: forewing ocherous brown in ground color (Fig. 1); phallus with seven cornuti (Fig. 4, arrows).

#### Description.

Head: Vertex and front white, tinged with pale ocherous yellow on cervical area; labial palpi white, first segment with dark brown scales dorsally, second segment somewhat tinged with pale ocherous brown, denser ventrally, third segment with broad pale ocherous brown ring at distal 1/4; antenna with scape white, covered with pale ocherous yellow scales on dorsal surface, pecten pale yellow, flagellum pale ocherous yellow, ringed with white; proboscis white.

Thorax: Tegula and mesonotum white, with three transverse pale ocherous brown bands; legs pale yellowish white, tibiae and tarsi pale ocherous brown on outer surface and pale ocherous brown and pale yellowish white on inner surface. Forewing (Fig. 1): length 5.5–7.0 mm (N = 5), about 3.3 × as long as wide, ocherous brown, slightly paler between CuP and dorsum; an N-shaped pinkish pattern running from basal 2/5 of costa diagonally outward to posterior angle of cell, obliquely to subapical part of costa, enlarged on costal margin, and diagonally narrowed to ventroapical part of termen; innermost stripe edged with two erect scale tufts, tufts black or ocherous brown on inner margin, angle between innermost and middle stripes diffused with blackish brown scales, and anteriorly with an erect pale yellowish white scale tuft, middle and outmost stripes both edged with erect scale tufts, tufts black or ocherous brown on outer margins; CuP with an erect scale tuft near distal 1/3; cilia with basal 3/5 pale ocherous brown, distal 2/5 pinkish white, edged with dark brown; ventral surface grayish brown. Hindwing (Fig. 1): dark gray; cilia yellowish gray.

Male genitalia (Fig. 4): Uncus broad at base, gradually narrowed to about 1/2, apical half parallel bilaterally, apex rounded; gnathos obtriangular, with minute granules on apical half; tegumen with nearly trapezoid posterior margins fused with uncus, anterior margin deeply emarginate; valva short and broad, sub-rectangular, heavily setose on triangular area apically; costa nearly straight; ventral margin triangularly concave inward beyond end of sacculus; sacculus narrow, slightly arched ventrally, distally with a triangular process directing downward; vinculum narrowly banded; saccus short, triangular, apex bluntly rounded; juxta obtrapezoidal, posterior margin straight, anterior margin concave mesially, lateral margins deeply emarginate at middle, forming two opposable triangular arms; phallus stout, distal 1/4 protuberant, extending to a sharp point; vesica with six irregularly-shaped, plate-like cornuti, and one small cornutus (Fig. 4, arrows).

Female genitalia (Fig. 6a): Papillae anales setose, broadly rounded posteriorly; apophyses posteriores about 2.5 times length of apophyses anteriores; eighth sternite with posterior margin straight, setose on posterior half, darkly pigmented mesolaterally, paler mesolongitudinally; antrum wide, membranous; ductus bursae narrow and sclerotized posteriorly, elongate, with undulating internal sclerotization anteriorly, bearing inception of ductus seminalis on anterior part; ductus bursae subspherical, with a spiculate inner wall; signum a transverse, dentate band, with a posteriorly projecting semicircular lobe.

#### Remarks.

*Lasiochira wuzhishanensis* Yin, sp. nov. is very similar in wing pattern to *L. jianfengensis* Yin, Wang and Park (Figs 1, 2), but differs by having juxta with posterior margin straight (Fig. 4); phallus with seven cornuti (Fig. 4, arrows); and corpus bursae small, nearly spherical in shape (Fig. 6a). *Lasiochira jianfengensis* has juxta with posterior margin emarginate mesially (Fig. 5); phallus with six cornuti (Fig. 5, arrows); and corpus bursae ovate in shape and larger (Fig. 7a).

#### Biology.

Unknown. Adults were attracted to white light in May.

#### Distribution.

Known only from the type locality (China: Hainan province).

#### Etymology.

The species epithet is derived from the name of the type locality, Wuzhishan National Nature Reserve.

### Lasiochira pallidiptera

Taxon classificationAnimaliaLepidopteraOecophoridae

Yin, Wang & Park, 2014

23C40F47-8678-5059-9006-F2B542FC4403

Figs 3 , 8 , 9

Lasiochira pallidiptera Yin, Wang & Park, 2014: 30 (Lasiochira; type locality: Mt. Godae-san, Yeoncheon-gun, Gyeongbuk province, Korea).

#### Material examined.

3 ♂♂, 1 ♀; China, Hubei province, Huanggang City, Yingshan County, Taohuachong Forest Park; alt. 700 m; 30°59'04"N, 115°56'15"E; 5 Jun. 2018; Zhengyong Wang leg.; YAH18154 ♂, YAH18157 ♂, YAH18160 ♀, YAH19075 ♂.

#### Remarks.

This species is recorded from China for the first time.

#### Biology.

Unknown. Adults were attracted to white light in June.

#### Distribution.

China (Hubei province), Korea (Central).

**Figure 10.**
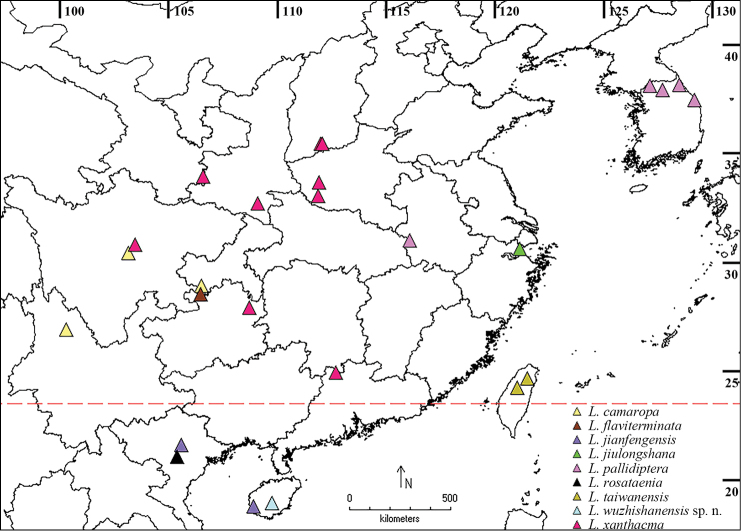
Distribution map of *Lasiochira* species.

## Supplementary Material

XML Treatment for Lasiochira

XML Treatment for Lasiochira wuzhishanensis

XML Treatment for Lasiochira pallidiptera
